# Nicorandil Attenuates LPS-Induced Acute Lung Injury by Pulmonary Endothelial Cell Protection via NF-*κ*B and MAPK Pathways

**DOI:** 10.1155/2019/4957646

**Published:** 2019-03-10

**Authors:** Mengyu He, Wen Shi, Min Yu, Xiang Li, Jian Xu, Jiali Zhu, Linling Jin, Weiping Xie, Hui Kong

**Affiliations:** Department of Respiratory and Critical Care Medicine, The First Affiliated Hospital of Nanjing Medical University, Nanjing, Jiangsu 210029, China

## Abstract

Acute lung injury (ALI) is a devastating critical disease characterized by diffuse inflammation and endothelial dysfunction. Increasing evidence, including from our laboratory, has revealed that the opening of ATP-sensitive potassium (K_ATP_) channels has promising anti-inflammation and endothelial protection activities in various disorders. However, the impacts of K_ATP_ channels on ALI remain obscure. In this study, we used nicorandil (Nico), a classic K_ATP_ channel opener, to investigate whether opening of K_ATP_ channels could alleviate ALI with an emphasis on human pulmonary artery endothelial cell (HPAEC) modulation. The results showed that Nico inhibited lipopolysaccharide- (LPS-) induced inflammatory response, protein accumulation, myeloperoxidase activity, and endothelial injury. In vitro, Nico reduced LPS-induced HPAEC apoptosis and the expression of cleaved-caspase-3, caspase-9, and CCAAT/enhancer-binding protein homologous protein (CHOP). Additionally, Nico inhibited inflammation by suppressing monocyte-endothelial adhesion and decreasing the expression of proinflammatory proteins. Moreover, Nico restored the expression and the distribution of adherens junction vascular endothelial- (VE-) cadherin. Further, Nico abolished the increase in intracellular reactive oxygen species (ROS) and the activation of NF-*κ*B and mitogen-activated protein kinase (MAPK) in HPAECs. Glibenclamide (Gli), a nonselective K_ATP_ channel blocker, abrogated the effects of Nico, implying that opening of K_ATP_ channels contributes to the relief of ALI. Together, our findings indicated that Nico alleviated LPS-induced ALI by protecting ECs function via preventing apoptosis, suppressing endothelial inflammation and reducing oxidative stress, which may be attributed to the inhibition of NF-*κ*B and MAPK signaling pathways.

## 1. Introduction

Acute lung injury (ALI) is a life-threatening disease with high incidence and mortality in critically ill patients [1]. Bacterial infections are the leading cause of ALI and may progress towards acute respiratory distress syndrome (ARDS), regarded as the severe form of ALI. It is generally agreed that the uncontrolled inflammation, endothelial injury, and oxidative damage participate in the pathogenesis of ALI, causing injured alveolar-capillary barrier, increased lung permeability, deficient gas exchange, and ultimately respiratory failure [2]. Although antibiotic therapy and intensive care are developing, the prognosis of ALI remains poor [3]. Herein, there is an urgent need for safe and effective therapeutic options in ALI treatment.

Recently, pulmonary endothelium has been considered as a key modulator and orchestrator of ALI. As a dynamic and active organ lining of the entire pulmonary vessel, adjacent endothelial cells (ECs) are tightly connected to each other via intercellular junctions, performing as a semipermeable barrier for the separation of pulmonary circulation from lung interstitium [4]. This barrier performs a regulatory role in lung homoeostasis by controlling the transport of fluids, proteins, and cells, as well as trafficking of inflammatory cells [5]. However, ECs are vulnerable to injury from noxious stimuli including circulating (e.g., lipopolysaccharide (LPS)), released (e.g. reactive oxygen species (ROS)), and physical factors, leading to barrier disruption and proinflammatory protein secretion [6]. Moreover, ECs play major roles in vascular remodeling by regulating the clotting system, vascular tone, and angiogenesis, which may determine the outcome of ALI [7]. Thus, the endothelial-targeted therapy may emerge as a promising strategy in ALI management.

ATP-sensitive potassium (K_ATP_) channels, first identified in cardiac tissue, are a unique link between cellular metabolic status and electrical activity [8]. Recently, nicorandil (Nico), a classic K_ATP_ channel opener (KCO), has been shown to exert strong endothelial protective and anti-inflammation potential. Recent evidence has indicated that Nico has therapeutic effects in pulmonary arterial hypertension (PAH) via attenuating vascular endothelial damage and inflammatory responses [9, 10]. Nico inhibited neutrophil recruitment in carrageenan-induced pleurisy [11], alleviated oxidative stress in cyclophosphamide-induced lung injury [12], and decreased paw edema induced by carrageenan [13]. Additionally, K_ATP_ channels have been found to be involved in numerous cellular processes, such as proliferation, apoptosis, migration, and angiogenesis. *In vitro*, Nico shows endothelial protective impacts when stimuli occur, such as hypoxia and high glucose [14, 15]. Although Nico has received increasing attention in the treatment of several pulmonary diseases, it remains unclear whether it has benefits in ALI therapy.

In the current study, a murine model of ALI induced by intratracheal LPS administration and cultured human pulmonary artery endothelial cells (HPAECs) were applied to determine the efficacy of Nico and its underlying potential cellular and molecular mechanisms.

## 2. Materials and Methods

### 2.1. Animal

Specific pathogen-free male C57B/L mice (20–22 g) were obtained from Cavans Laboratories (Changzhou, China). The mice were kept in a temperature-controlled room under a 12 hr dark/light cycle and were provided with food and water *ad libitum*. All experimental protocols were performed according to the guidelines of the National Institutes of Health and Nanjing Medical University, and all procedures were approved by the Institutional Animal Care and Use Committee of Nanjing Medical University (Nanjing, China).

### 2.2. LPS-Induced ALI in Mice

**Figure 1:**
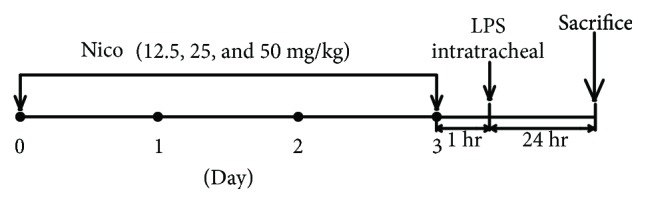
C57B/L mice were treated with Nico (12.5, 25, and 50 mg/kg) for 3 consecutive days by gavage. On day 3, Nico was given at 1 hr before intratracheal instillation of LPS or saline administration. Mice were sacrificed at 24 hr after LPS administration. Nico: nicorandil.

After anesthesia, the mice received an intratracheal instillation of LPS (5 mg/kg, Sigma-Aldrich, St. Louis, MO, USA) dissolved in 50 *μ*L of normal saline or saline alone as the control. To investigate the preventive effects, the mice were treated with Nico (12.5, 25, and 50 mg/kg) (Sigma-Aldrich) for 3 consecutive days by gavage. On day 3, the mice were administrated with Nico at 1 hr before LPS challenge or were treated with Nico (50 mg/kg) at 1 hr before saline administration (*n* = 6 − 8 for each group). The mice were sacrificed at 24 hr after LPS administration (Figure 1). Bronchoalveolar lavage fluid (BALF) was collected by lavage with ice-cold phosphate-bufferedsaline (PBS, 400 *μ*L for 3 times; 85–90% of the lavage volume was recovered) via a tracheal catheter. After centrifugation, the supernatants were frozen at -80°C for further experiments. The lung lobes were collected for further analysis.

### 2.3. Lung Histology

The lung tissues were placed in 4% paraformaldehyde fixative for paraffin embedding. Then, a series of microsections (5 *μ*m) were cut with a microtome and stained with hematoxylin and eosin (H&E). For each section, at least eight random areas were selected by a light microscope (DM2500, Leica, Wetzlar, Germany). The degree of lung injury was assessed by a semiquantitative scoring system.

### 2.4. Measurement of Protein Content and Myeloperoxidase (MPO)

Protein concentrations in BALF were determined by BCA Protein Assay Kit (Beyotime, Nantong, China). MPO activities were measured by a commercial test kit (Jiancheng Bioengineering Institute, Nanjing, China) in both BALF and lung homogenate. All the procedures were performed according to the manufacturer's protocol.

### 2.5. Cell Culture

HPAECs were purchased from Sciencell Research Laboratories (Carlsbad, CA, USA). HPAECs were cultured in endothelial cell medium-2 (ECM-2) (Sciencell, USA), consisting of 5% fetal bovine serum (FBS), 1% penicillin, streptomycin, and 1% endothelial cell growth factors. Cells were used in their fourth to sixth passages. After starvation for 6 hr, cells were pretreated with Nico for 1 hr before stimulation with LPS (1 *μ*g/mL) for indicated time intervals. To investigate the interaction between KCOs and K_ATP_ blockers on HPAECs, glibenclamide (Gli), a nonselective K_ATP_ blocker, was added 30 min prior to the addition of other drugs at the concentration of 10 *μ*mol/L. Gli was purchased from Sigma-Aldrich. Nico and Gli were diluted with dimethyl sulphoxide (DMSO), and LPS was diluted with double-distilled H_2_O. The concentration of DMSO was controlled below 0.1% (vol/vol) and did not decrease the biological viability of the cells. The human monocytic leukemia cell line U-937 cells were purchased from the Institute of Biochemistry and Cell Biology, Chinese Academy of Sciences (Shanghai, China). The U-937 cells were cultured in RPMI 1640 (Gibco-BRL, Grand Island, USA) containing 10% FBS. HPAECs and U-937 cells were incubated at 37°C in a humidified incubator with 5% CO_2_.

### 2.6. Cell Viability Assay

Cell counting kit-8 (CCK8) assay was conducted to analyze the cytotoxicity of Nico on HPAECs. HPAECs were seeded into a 96-well plate at a density of 10,000/well overnight. Then, cells were treated with Nico (0.1, 1, 10, and 100 *μ*M) for 24 hr. Cell viability was performed with CCK-8 kits (Dojindo Molecular Technologies, Kumamoto, Japan) according to the manufacturer's protocol. Absorbency was measured at 450 nm by a microplate reader (Thermo Scientific, Sunnyvale, CA, USA).

### 2.7. Fluorescent Staining of Cells with Hoechst 33342

Hoechst 33342 staining was performed to detect the apoptotic rate of HPAECs. Briefly, cells were cultured in 24-well plates and treated at ~80% confluency. After exposure to LPS for 24 hr, the cells were washed with PBS prior to 30 min staining with Hoechst 33342 (10 mg/mL) (Beyotime). Five fields were randomly selected from each dish under fluorescence microscopy (DM2500, Leica, Wetzlar, Germany), and at least 3 dishes were counted per treatment. The percentage of apoptosis was calculated by counting.

### 2.8. cDNA Synthesis and Quantitative Polymerase Chain Reaction (qRT-PCR)

HPAECs were seeded into 12-well plates and stimulated with LPS in the presence or absence of Nico and Gli for 6 hr. Total RNA was extracted from HPAECs with TRIzol reagent (Gibco-BRL, Grand Island, New York, USA). Reverse transcription was performed with 500 ng of total RNA with SYBR®Premix Ex Taq™ (TaKaRa, Shiga, Japan). Two-step real-time RT-PCR was used to perform relative quantification of mRNA. qRT-PCR was performed using primers selected for TNF-*α*, COX-2, and *β*-actin (Table 1). The ΔΔCt method was used to quantify mRNA expression relative to *β*-actin.

### 2.9. Monocyte-Endothelial Cell Adhesion Assay

The adherence of U-937 cells to activated HPAECs was evaluated by an established coculture system as previous described with minor modifications [16, 17]. U-937 cells were labeled with 4 ng/mL calcein-AM (BD Biosciences) for 30 min at 37°C. After pretreatment with Nico for 1 hr in the presence or absence of Gli, HPAECs were stimulated with LPS for 12 hr. LPS-treated ECs were coincubated with U-937 cells (2 × 10^5^ cells/mL) at 37°C for 1 hr. Nonadherent monocytes were removed by gently washing with PBS. 4,6-Diamido-2-phenylindole hydrochloride (DAPI) was used for nuclei staining. The number of adherent monocytes in five random areas of each well was counted under a fluorescence microscope (Leica, Wetzlar, Germany).

### 2.10. Immunofluorescence Staining

HPAECs were cultured into 24-well plates for 24 hr before exposure to Nico or Gli with LPS. After being washed with PBS, cells were fixed with 4% paraformaldehyde and blocked with 5% BSA. Then, HPAECs were incubated with vascular endothelial- (VE-) cadherin (1 : 200), manganese superoxide dismutase (MnSOD) (1 : 200), Nox4 (1 : 500) (Abcam, Cambridge, UK), and anti-NF-*κ*B p65 (1 : 100, Cell Signaling Technology, Danvers, MA, USA) at 4°C overnight. Alexa Fluor 555 donkey anti-rabbit IgG (Thermo Fisher Scientific, Waltham, USA) was used to detect antibody binding. DAPI was used for nuclei staining. Cells were visualized by an inverted fluorescent microscope (Leica, Wetzlar, Germany).

### 2.11. ELISA

Concentrations of TNF-*α* and IL-1*β* in the lung tissues were measured with murine cytokine-specific ELISA kits (R&D Systems, Abingdon, UK). In brief, the lung homogenate was centrifuged at 3000 rpm for 20 min at 4°C, and the supernatant was analyzed using ELISA kits according to the manufacturer's instructions.

The level of NADPH oxidase 4 (Nox4) in HPAECs was measured. The cells were treated as described above, and the ELISA was conducted according to the manufacturer's protocols (Yifeixue Biotechnology, Nanjing, China).

### 2.12. Determination of Intracellular ROS Production

Intracellular ROS were detected using the 2′,7′-dichlorofluorescin diacetate (DCFH-DA) assay with a laser scanning confocal microscope (Leica, Wetzlar, Germany). Briefly, HPAECs were seeded into each well of a 24-well plate, cultured for 24 hr, and pretreated with Nico (100 *μ*M) with or without Gli (10 *μ*M) before exposure to LPS (1 *μ*g/mL) for 1 hr. The cells were then incubated with 10 *μ*M DCFH-DA for 30 min at 37°C in the dark. After being fixed with 4% paraformaldehyde for 10 min, the cells were washed three times with PBS before being photographed. For each culture, a minimum of 5 random fields was captured with an excitation setting of 488 nm.

### 2.13. Western Blotting Analysis

The protein of lung tissues and HPAECs was collected in a lysis buffer (Beyotime), and centrifuged for 15 min at 12,000 rpm at 4°C. Protein concentrations were detected using a BCA Protein Assay Kit (Beyotime). The samples were separated by SDS-polyacrylamide gel electrophoresis (SDS-PAGE) and transferred onto polyvinylidene difluoride (PVDF) membranes (Millipore, Billerica, USA). The membranes were blocked with Tris-buffered saline containing 0.05% Tween 20 (TBST) and 5% nonfat milk for 1 hr at room temperature. Then, the transferred membranes were incubated with primary antibodies against Kir6.1 (Alomone Labs, Jerusalem, Israel), Kir6.2 (Abcam), NF-*κ*B p-p65/p65, p-i*κ*B-*α*/i*κ*B-*α*, p-p38/p38, p-ERK/ERK, p-JNK/JNK, intercellular adhesion molecule-1 (ICAM-1), cleaved-caspase-3 (c-caspase-3), caspase-9 (1 : 1000, Cell Signaling Technology), endothelial nitric oxide synthase (eNOS) (1 : 1000, Santa Cruz), inducible nitric oxide synthase (iNOS) (1 : 1000, Millipore), CCAAT/enhancer-binding protein homologous protein (CHOP), vascular cell adhesion molecule-1 (VCAM-1), VE-cadherin, Nox4 (1 : 1000), MnSOD (1 : 5000, Abcam), and *β*-actin (1 : 5000, Proteintech, Rosemont, USA) overnight. The blot was incubated with the appropriate horseradish-peroxidase- (HRP-) conjugated goat anti-mouse antibody and goat anti-rabbit antibody (1 : 10,000, Proteintech) for 1 hr at room temperature. The signals were visualized using an enhanced chemiluminescence (ECL) reagent kit (Thermo Fisher Scientific, Waltham, USA) and analyzed with Bio-Rad Gel Doc/ChemiDoc Imaging System and Quantity One software (Bio-Rad, Hercules, CA, USA).

### 2.14. Statistical Analysis

All data were presented as mean ± SEM. Comparisons between controls and samples treated with different drugs were determined by one-way analysis of variance (ANOVA). Least significant difference (LSD) post hoc tests were used to assess significant effects. *P* < 0.05 was considered statistically significant.

## 3. Results

### 3.1. Nico Attenuated LPS-Induced Lung Injury

**Figure 2:**
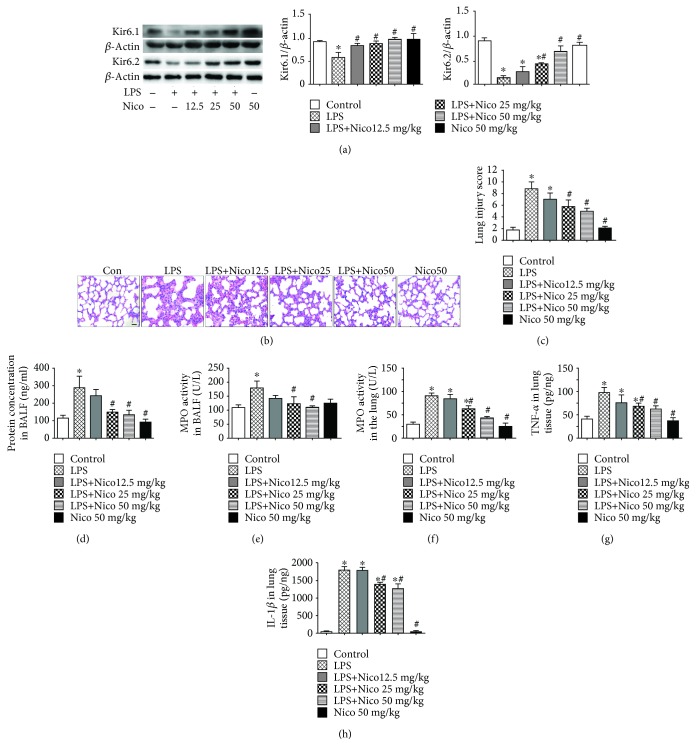
Nico ameliorated LPS-induced ALI and inflammation. (a) Nico increased LPS-induced Kir6.1 and Kir6.2 downregulation in the lung. (b, c) Lung sections stained with H&E showed severe injury in the LPS group which was attenuated by Nico pretreatment. The data revealed a high score for the LPS-treated group which was decreased in the Nico-pretreated group. (d) Nico pretreatment significantly reduced LPS-induced protein leakage in BALF. (e, f) Nico alleviated LPS-induced increments of MPO activities in BALF and lung homogenate. (g, h) Nico prevented the production of TNF-*α* and IL-1*β* in lung homogenate. Data were shown as mean ± SEM (*n* = 6 − 8). Statistically significant differences: ^∗^*P* < 0.05 versus the control group; #*P* < 0.05 versus the LPS group. Scale bar: 50 *μ*m.

Lung tissues of mice were harvested to investigate the effects of LPS and Nico on K_ATP_ channel activation. The expression of K_ATP_ channel subunits (Kir6.1 and Kir6.2) was examined by western blotting. LPS challenge led to significant downregulation of Kir6.1 and Kir6.2 in the lungs (Figure 2(a)). However, these effects of LPS were significantly inhibited by pretreatment with Nico in a concentration-dependent way.

To detect the role of Nico in LPS-mediated lung injury, lung sections stained with H&E were used for detecting histological changes at 24 hr after LPS challenge. Compared with the control group, the mice treated with LPS showed increased severity of lung injury correlated with greater diffuse alveolar damage, alveolar wall thickening (Figures 2(b) and 2(c)), and elevated BALF protein (Figure 2(d)). Pretreatment with Nico (25 mg/kg and 50 mg/kg) alleviated LPS-induced lung injury (Figures 2(b) and 2(c)) and significantly suppressed the increase of protein level in BALF after LPS challenge (*P* < 0.05) (Figure 2(d)), while 12.5 mg/kg Nico treatment did not result in such significant changes.

### 3.2. Nico Ameliorated LPS-Induced Lung Inflammation

MPO activities in BALF and lung homogenate were detected to assess neutrophil infiltration in LPS-induced ALI. Compared with the LPS group, administration of Nico (25 mg/kg and 50 mg/kg) induced remarkable decreases in MPO activities (*P* < 0.05), while 12.5 mg/kg Nico (12.5 mg/kg) did not cause notable decreases (Figures 2(e) and 2(f)).

TNF-*α* and IL-1*β* were analyzed to investigate the inflammatory response. As indicated in Figures 2(g) and 2(h), Nico (25 mg/kg and 50 mg/kg) significantly ameliorated LPS-induced increased levels of TNF-*α* and IL-1*β* in lung homogenates (*P* < 0.05).

### 3.3. Nico Alleviated EC Injury in LPS-Challenged Mice

**Figure 3:**
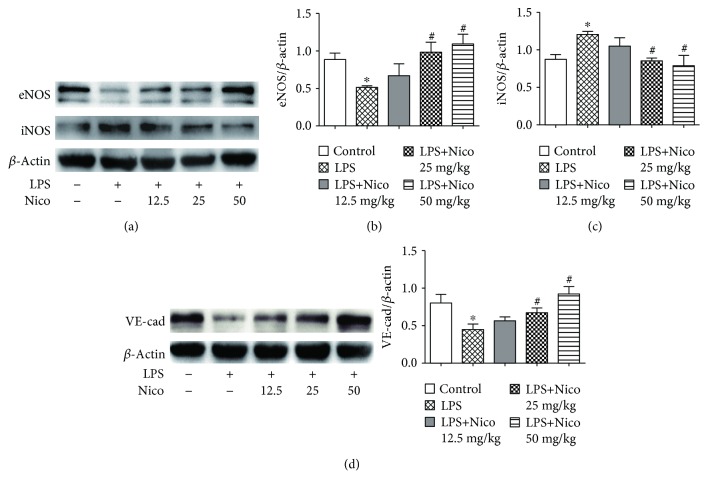
Nico alleviated EC injury in LPS-challenged mice. (a–d) Treatment with Nico preserved the expression of endothelial-related proteins eNOS and VE-cadherin and restrained the expression of iNOS *in vivo*. Data were shown as mean ± SEM (*n* = 3 − 4). Statistically significant differences: ^∗^*P* < 0.05 versus the control group; ^#^*P* < 0.05 versus the LPS group.

To investigate the effects of Nico on LPS-induced EC injury in ALI, endothelial related proteins eNOS, iNOS, and VE-cadherin were detected. Although the expression of iNOS was upregulated in the LPS group, Nico dose-dependently prevented the change (*P* < 0.05) (Figures 3(a) and 3(c)). As shown in Figures 3(a) and 3(d), western blotting analysis revealed significant downregulation of eNOS and VE-cadherin in the lungs of LPS-challenged mice. Nico at the concentration of 25 mg/kg and 50 mg/kg alleviated LPS-decreased expression of VE-cadherin and eNOS in the lungs of mice (*P* < 0.05).

### 3.4. Nico Decreased LPS-Induced HPAEC Apoptosis

**Figure 4:**
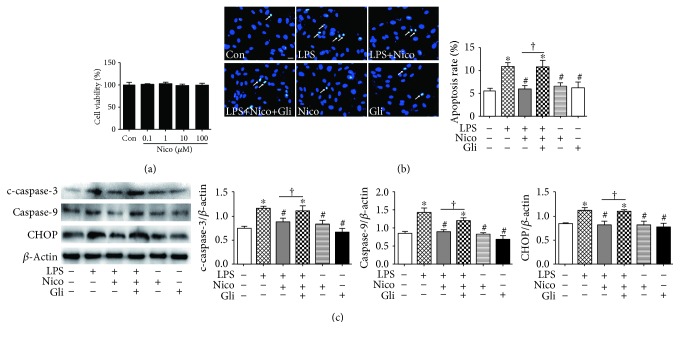
Nico decreased LPS-induced HPAEC apoptosis. (a) Effects of Nico on the viabilities of HPAECs were assessed by CCK-8. (b) Hoechst 33342 staining showed that Nico decreased HPAEC apoptosis induced by LPS, and Gli abolished the effects. (c) Nico pretreatment markedly inhibited the expression of c-caspase-3, caspase-9, and CHOP in HPAECs stimulated with LPS, and Gli abolished the effects. Data were shown as mean ± SEM of at least three independent experiments performed in triplicate. Statistically significant differences: ^∗^*P* < 0.05 versus the control group; ^#^*P* < 0.05 versus the LPS group; ^†^*P* < 0.05 versus the LPS+Nico group. Scale bar: 25 *μ*m.

To evaluate the possible cytotoxic potential of Nico (0.1, 1, 10, and 100 *μ*M), cellular viability was determined on HPAECs. The results showed that there was no cellular toxicity induced by Nico between the concentrations of 0.1 *μ*M and 100 *μ*M (Figure 4(a)).

As indicated in Figure 4(b), apoptotic nuclei with chromatin condensation and nuclear fragmentation were more easy to be dyed and showed brilliant blue fluorescence, while nonapoptotic nuclei showed light blue fluorescence. Statistical analysis demonstrated that pretreatment with Nico (100 *μ*M) alleviated LPS-induced HPAEC apoptosis. Moreover, the inhibitory effect of Nico on LPS-induced cell apoptosis could be abolished by Gli (*P* < 0.05). The findings were further confirmed by western blotting analysis. As shown in Figure 4(c), Nico potently decreased LPS-induced expression of c-caspase-3 and caspase-9, critical executioners of apoptosis, and suppressed the proapoptotic transcription factor CHOP. Gli blocked the beneficial effects of Nico (*P* < 0.05). Nico and Gli themselves did not trigger HPAEC apoptosis.

### 3.5. Nico Restrained LPS-Induced Proinflammatory Factor Expression in HPAEC

**Figure 5:**
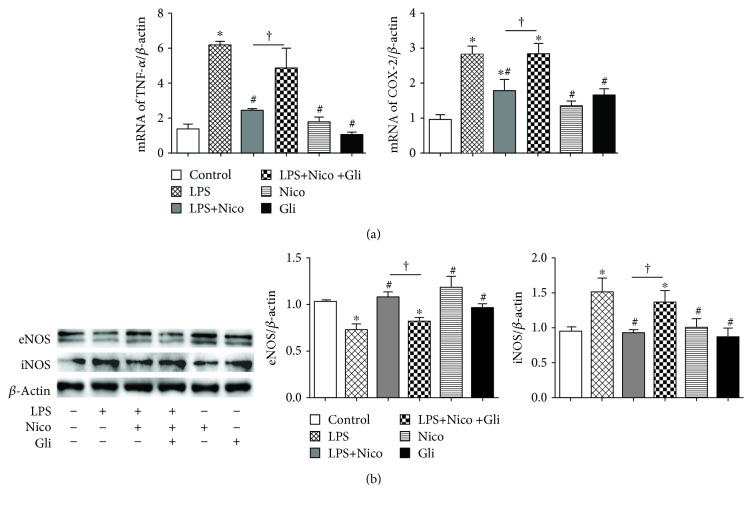
Treatment with Nico restrained the proinflammatory factors induced by LPS in HPAECs. (a, b) Nico decreased the LPS-induced expression of TNF-*α* and COX-2 in the mRNA level and the expression of iNOS in the protein level, whereas it increased LPS-induced downregulation of eNOS in the protein level. The alternations originated from Nico could be abolished by Gli. Data were shown as mean ± SEM of at least three independent experiments performed in triplicate. Statistically significant differences: ^∗^*P* < 0.05 versus the control group; ^#^*P* < 0.05 versus the LPS group; ^†^*P* < 0.05 versus the LPS+Nico group.

Pulmonary ECs have been reported to release cytokines and adhesion molecules, leading to an inflammatory process and activation of leukocytes in the pathogenesis of ALI [18]. To further substantiate the anti-inflammatory mechanism of Nico, we studied its effects on LPS-induced expression of proinflammatory mediators in HPAECs. The results showed that Nico markedly blocked LPS-induced upregulation of TNF-*α* and partially decreased the LPS-induced COX-2 expression in mRNA level (*P* < 0.05) (Figure 5(a)). Additionally, Nico suppressed the LPS-induced increased expression of iNOS, while Nico recovered LPS-downregulated expression of eNOS in the protein level (*P* < 0.05) (Figure 5(b)). Pretreatment of Gli, a nonselective K_ATP_ blocker, eliminated the inhibitory effects of Nico on LPS-induced inflammatory responses (*P* < 0.05) (Figure 5(b)). Nico itself could slightly induce the expression of eNOS, even though no statistical significance was observed (*P* = 0.072).

### 3.6. Nico Suppressed LPS-Induced Adhesion Molecule Expression and Adhesion between Endothelial Cells and Monocytic Cells

**Figure 6:**
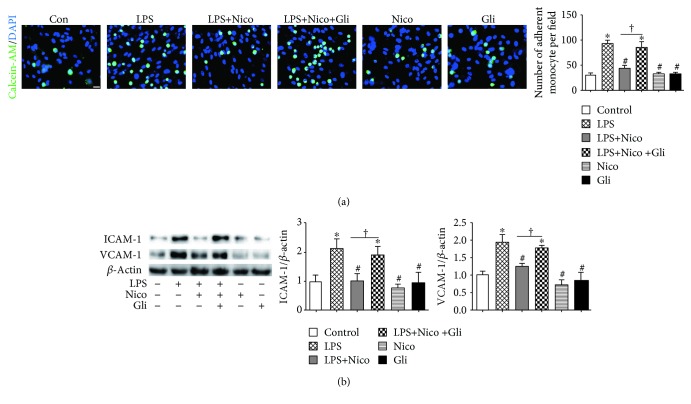
Nico suppressed HPAEC-mediated monocyte-endothelial adhesion. (a) Nico pretreatment suppressed monocyte-endothelial adhesion induced by LPS stimulation. Gli abolished the effect of Nico. (b) Nico prevented the overexpression of ICAM-1 and VCAM-1 in the LPS-treated group. Gli abolished the effects of Nico. Data were shown as mean ± SEM of at least three independent experiments performed in triplicate. Statistically significant differences: ^∗^*P* < 0.05 versus the control group; ^#^*P* < 0.05 versus the LPS group; ^†^*P* < 0.05 versus the LPS+Nico group. Scale bar: 25 *μ*m.

HPAECs were treated with LPS for 12 hr, and then monocyte adhesion to ECs was determined. Compared to the untreated control cells, monocytic adhesion to LPS-stimulated ECs was significantly increased (*P* < 0.05) (Figure 6(a)). Nico pretreatment resulted in reduced adhesion of monocytes to HPAECs compared to the LPS-treated cells (*P* < 0.05) (Figure 6(a)). Moreover, Gli inhibited Nico-alleviated adhesion of monocytes to HPAECs (*P* < 0.05) (Figure 6(a)).

Expression of adhesion molecules on the surface of ECs is important for the association of monocytes in circulation. We detected the effects of Nico in regulating the expression of ICAM-1 and VCAM-1, two critical adhesion molecules, in HPAECs in response to LPS. Upon stimulation with LPS, the expression of ICAM-1 and VCAM-1 was significantly increased in HPAECs (*P* < 0.05) (Figure 6(b)). Pretreatment with Nico showed a reduction of ICAM-1 and VCAM-1 expression (*P* < 0.05) (Figure 6(b)). The addition of Gli abolished the effects of Nico (*P* < 0.05) (Figure 6(b)).

### 3.7. Nico Recovered LPS-Induced HPAEC Loss of VE-Cadherin

**Figure 7:**
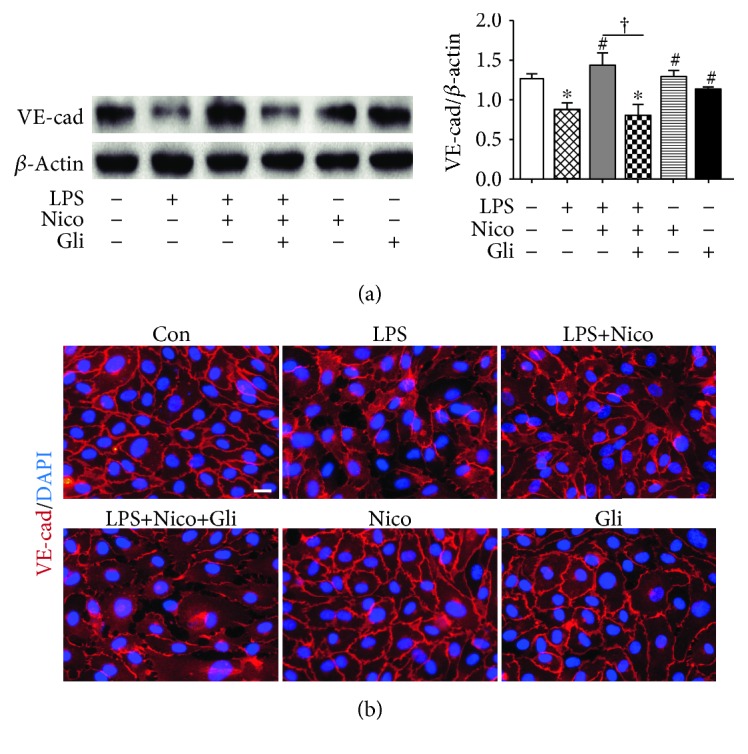
Nico alleviated LPS-induced loss of VE-cadherin in HPAECs. (a) Western blotting showed that Nico pretreatment protected the reduced expression of VE-cadherin. (b) Immunofluorescence staining revealed that Nico pretreatment partially recovered the internalization of VE-cadherin caused by LPS. Gli could reverse the alternations elicited by Nico. Data were shown as mean ± SEM of at least three independent experiments performed in triplicate. Statistically significant differences: ^∗^*P* < 0.05 versus the control group; ^#^*P* < 0.05 versus the LPS group; ^†^*P* < 0.05 versus the LPS+Nico group. Scale bar: 25 *μ*m.

VE-cadherin is the major component for the adherens junctions (AJs) between ECs, which plays a critical role in controlling vascular endothelial function and endothelial permeability [19]. Herein, the expression and distribution of VE-cadherin were investigated. The results from western blotting analysis showed that the expression of VE-cadherin was notably decreased by LPS challenge for 24 hr. Pretreatment with Nico recovered the LPS-induced downregulation of VE-cadherin, while Gli could abolish the effects (*P* < 0.05) (Figure 7(a)). Besides, immunofluorescence staining confirmed that Nico protected HPAECs from LPS-induced loss of VE-cadherin at cell-cell junctions (Figure 7(b)). Gli abolished Nico-induced beneficial effects in HPAECs.

### 3.8. Nico Diminished LPS-Induced Oxidative Stress in HPAEC

**Figure 8:**
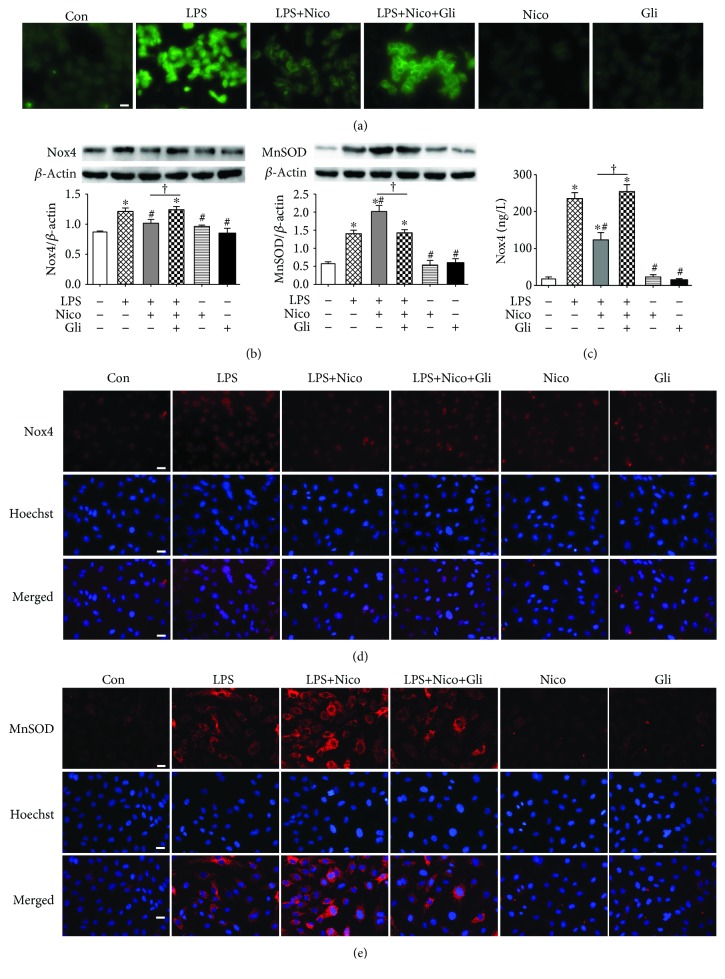
Treatment with Nico attenuated oxidative stress induced by LPS. (a) Intracellular ROS was evaluated by DCFH-DA fluorescence (green) imaging which revealed that Nico reduced the ROS production induced by LPS and Gli blunted the alternation. (b, d) Western blotting analysis and immunofluorescence staining showed that pretreatment with Nico decreased the overexpression of Nox4 after LPS stimulation. (c) ELISA showed that Nico attenuated the activation of Nox4. The effects could be blocked by Gli. (b, e) Western blotting and immunofluorescence staining revealed that pretreatment with Nico induced a higher expression of MnSOD compared to the LPS group. Data were shown as mean ± SEM of at least three independent experiments performed in triplicate. Statistically significant differences: ^∗^*P* < 0.05 versus the control group; ^#^*P* < 0.05 versus the LPS group; ^†^*P* < 0.05 versus the LPS+Nico group. Scale bar: 25 *μ*m.

Previous studies have suggested a strong association with oxidative stress and ALI. As shown in Figure 8(a), LPS apparently promoted ROS production, while pretreatment with Nico decreased the intracellular ROS level. Gli inhibited the effect of Nico on regulating ROS production.

We further investigated the mechanisms by which Nico blocked oxidative stress. As the most ubiquitous of NAD(P)H oxidases, Nox4 is a major source of LPS-induced ROS generation in ECs [20]. Since Nico is known to primarily bind to mitochondrial K_ATP_ channel, we hypothesize that Nico may enhance mitochondrial antioxidative activity. Thus, MnSOD as a major antioxidant in mitochondria was detected. As shown in Figures 8(b) and 8(c), we found that the Nox4 level was significantly decreased after Nico treatment compared with the LPS group by the methods of western blotting and ELISA (*P* < 0.05). Additionally, LPS led to increased expression of MnSOD compared to the control group (*P* < 0.05). However, Nico pretreatment induced a higher expression of MnSOD compared to the LPS group (Figure 8(b)). The results were further confirmed by immunofluorescence staining (Figures 8(d) and 8(e)). The data suggested that Nico may prevent LPS-induced oxidative stress by regulating the key antioxidant systems in mitochondria.

### 3.9. Nico Suppressed LPS-Induced Activation of NF-*κ*B and MAPK Pathways in HPAEC

**Figure 9:**
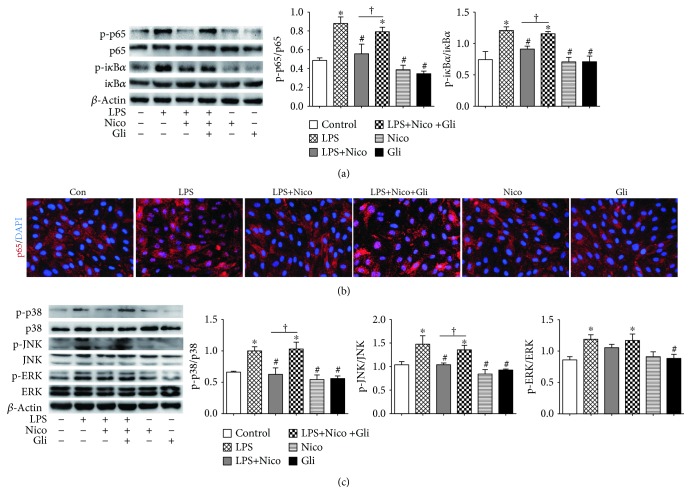
Effects of Nico on NF-*κ*B and MAPK activation in LPS-challenged HPAECs. (a) Nico significantly suppressed LPS-induced NF-*κ*B p65 and i*κ*B*α* phosphorylation. (b) Nico notably suppressed nuclear translocation of p65 in LPS-treated HPAECs. (c) Nico decreased LPS-induced p38 and p-JNK phosphorylation, but not ERK phosphorylation. Gli blunted the effects of Nico on NF-*κ*B and MAPK pathways. Data were shown as mean ± SEM of at least three independent experiments performed in triplicate. Statistically significant differences: ^∗^*P* < 0.05 versus the control group; ^#^*P* < 0.05 versus the LPS group; ^†^*P* < 0.05 versus the LPS+Nico group. Scar bar: 25 *μ*m.

It is widely accepted that NF-*κ*B pathway is crucial for LPS-induced inflammation in pulmonary ECs. Therefore, the phosphorylation of p65 and i*κ*B-*α* was examined. The data confirmed the effects of LPS on activating the NF-*κ*B p65 pathway in HPAECs within 30 min after stimulation. The activation was suppressed in the Nico-pretreated group (*P* < 0.05) (Figure 9(a)). Additionally, immunofluorescence staining showed that Nico inhibited LPS-induced NF-*κ*B p65 nuclear translocation (Figure 9(b)). Pretreatment with Gli could block the alternation elicited by Nico.

The activation of MAPKs is involved in the regulation of oxidative stress and inflammatory responses. As shown in Figure 9(c), LPS trigged significant activation of p38, JNK, and ERK within 30 min after stimulation (*P* < 0.05) (Figure 9(c)). Pretreatment of Nico could blunt the activation of p38 and JNK induced by LPS (*P* < 0.05), but not the activation of ERK (Figure 9(c)). Gli could reverse the suppression elicited by Nico, while no alternation was observed with Nico and Gli alone.

## 4. Discussion

It is well-known that ALI is featured as loss of alveolar-capillary membrane integrity, sustained inflammation, and excessive oxidative stress. As key components of alveolar-capillary barrier, lung ECs might be the first target of circulating soluble factors and inflammatory cells. The apoptosis of ECs causes loss of endothelial integrity, leading to increased lung microvascular permeability, alveolar edema, and leukocyte extravasations during ALI [2]. Mounting studies have confirmed that LPS treatment induces EC apoptosis. In the present study, exposure to LPS increased EC apoptosis, with the enhanced expression of c-caspase-3 and caspase-9, two critical proteases in programmed cell death, and CHOP, one vital transcription factor in endoplasmic reticulum stress- (ERS-) induced apoptosis. Activation of K_ATP_ channels by Nico blunted LPS-induced HPAECs apoptosis. Recently, CHOP has been regarded as a multifunctional molecule involved in apoptosis as well as in metabolic and inflammatory processes [21]. Dong et al. showed that the opening of K_ATP_ channels attenuated astrocyte injury and inflammation via suppressing ERS including the expression of CHOP [22]. Sargsyan et al. reported that activation of K_ATP_ channels improved *β*-cell function via reducing the expression of CHOP [23]. Our data showed that Nico decreased LPS-induced expression of CHOP, indicating that ERS may be involved in the protective effects of KCOs.

Persistent inflammation is another feature of ALI, characterized by inappropriate activity of inflammatory cells and substantial release of inflammatory mediators. In response to LPS, pulmonary endothelium becomes activated resulting in increased expression of adhesion molecules, excessive production of cytokines, and uncontrolled interaction with inflammatory cells [24]. In our *in vivo* study, the activation of K_ATP_ channels by Nico decreased LPS-induced cytokine release and effectively suppressed inflammatory cell infiltration. In our *in vitro* study, it showed that Nico pretreatment reduced the increased expression of inflammatory cytokines induced by LPS, whereas these effects partially disappeared upon treatment with the K_ATP_ channel inhibitor. Moreover, adhesion molecules play vital roles in regulating recruitment of leukocytes into the endothelium [25]. Our findings indicated that K_ATP_ channel could regulate the expression of adhesion molecules, as well as monocyte-endothelial adhesions.

Uncontrolled oxidative stress contributes to the development of ALI. LPS-induced overproduction of ROS, such as superoxide anion and hydrogen peroxide, can exacerbate tissue damage and inflammatory response [26]. Previous evidence has revealed that several antioxidant agents, such as vitamin D [27], propofol [28], and olmesartan [29], prevent ECs from injury induced by excessive oxidative stress. Thus, inhibition of oxidative stress may be one of the mechanisms to alleviate ALI. In ECs, Nox4 is a highly expressed NADPH oxidase among seven homologs (Nox1, Nox3, Nox4, Nox5, Duox1, and Duox2) of gp91phox (Nox2) which can directly affect the production of inflammatory cytokines [30]. Our results showed that LPS notably enhanced the production of ROS with an elevation of Nox4 expression, whereas Nico attenuated the oxidative stress in HPAECs. Interestingly, it showed that LPS upregulated the expression of MnSOD, one of the critical antioxidant enzymes, which may be a possible self-adjusting mechanism of HPAECs to withstand oxidative stress. It is generally accepted that LPS induces ROS production, which activates ECs causing overexpression of proinflammatory factors. However, these proinflammatory proteins may in turn exacerbate inflammatory responses, either direct or indirect participation in oxidative stress. Herein, there is a vicious feedback loop occurring due to cytotoxic activities of ROS during ALI. Our findings suggested that the anti-inflammation effects of K_ATP_ channel activation may be partly due to its ROS scavenging activity, leading to a reduction in LPS-induced oxidative damage.

It is recognized that AJs are essential parts in paracellular pathways, and their disruption leads to interendothelial adhesion breakdown and endothelial barrier hyperpermeability. VE-cadherin is a major component of AJs and is used to correlate the destruction or enhancement of the endothelial monolayer barrier function [31]. LPS has been shown to induce the abnormal distribution and decrease expression of VE-cadherin [32]. Correspondingly, we found that LPS significantly decreased the expression of VE-cadherin in vivo and in vitro; besides, activation of K_ATP_ channels may maintain the barrier function of ECs by restoring interendothelial AJs. A previous study showed that Nico preserved endothelial junctions of VE-cadherin by decreasing endothelin-1 in acute myocardial infarction and reperfusion model [33]. Additionally, VE-cadherin is a calcium-dependent cell-cell adhesion molecule, while it is widely accepted that KCOs participate in calcium signaling modulation [34, 35]. Thus, it is possible that inflammatory responses and calcium signaling may be involved in the regulation of K_ATP_ channels in AJ expression and distribution.

Both NF-*κ*B and MAPK pathways are involved in the pathology of ALI. When stimulated with LPS or ROS, NF-*κ*B signaling cascade becomes activated by degradation and phosphorylation of i*κ*B-*α* as well as nuclear translocation and phosphorylation of NF-*κ*B p65 [36, 37]. Additionally, inhibition of MAPK pathways, including p38, ERK, and JNK, can alleviate the transcription of proinflammatory mediators and oxidative stress [38]. Studies have shown that activation of K_ATP_ channels prevents ECs from hypoxia-induced apoptosis through p38 MAPK and NF-*κ*B pathways [14, 39] and ameliorated excess oxidative stress through NF-*κ*B signaling [40]. In terms of the current study, it suggested that the endothelial protection of K_ATP_ channels is related to NF-*κ*B and MAPK signaling pathways.

Our findings have confirmed that Nico suppresses LPS-induced ALI via endothelial protective effects. However, a previous study showed that endothelium may not be the target for KCOs to exert vasorelaxant effects [41]; accumulating data, including ours, have shown that K_ATP_ channels are critical in regulating EC functions to shear stress and arterial vasomotion [42, 43]. In mice, knockout of endothelial specific Kir6.1 leads to impaired vasorelaxation during hypoxia in the coronary circulation [44]. In HPAECs, KCOs are capable of reducing endothelial apoptosis, promoting the release of nitrogen oxide and preventing EC dysfunction from ischemia and hypoxia, which could be blocked by K_ATP_ channel blockers [9, 45, 46]. Although the present study showed that K_ATP_ channels played important roles in regulating EC functions, the mouse model with knockout of endothelial specific K_ATP_ channels may be used for further studies to demonstrate the protective effects of K_ATP_ channels on ECs in ALI.

## 5. Conclusions

The present study suggested that Nico attenuated LPS-induced ALI through preventing EC apoptosis, decreasing oxidative stress, and suppressing endothelial-mediated inflammation. These protective effects of Nico may be attributed to the activation of K_ATP_ channels and its downstream NF-*κ*B and MAPK signaling pathways. Taken together, our results provide direct evidence that Nico might be a candidate for the adjuvant therapy for ALI patients.

## Figures and Tables

**Table 1:** Primers for qRT-PCR.

Target gene	Primer sequence
TNF-*α*	F: CGAAGTGGTGGTCTTGTTGCT
	R: CCTGCCCCAATCCCTTTATTA

COX-2	F: TGTATGAGTGTGGGATTTGA
	R: TGTGTTTGGAGTGGGTTT

*β*-Actin	F: GACATCCGCAAAGACCTG
	R: GGAAGGTGGACAGCGAG

Abbreviations: TNF-*α*: tumor necrosis factor-*α*; COX-2: cyclooxygenase-2; F: forward; R: reverse.

## Data Availability

The data used to support the findings of this study are available from the corresponding author upon request.
